# Improving Blood Pressure Control in a Large Multiethnic California Population Through Changes in Health Care Delivery, 2004–2012

**DOI:** 10.5888/pcd11.140173

**Published:** 2014-10-30

**Authors:** Kate M. Shaw, Joel Handler, Hilary K. Wall, Michael H. Kanter

**Affiliations:** Author Affiliations: Joel Handler, Michael H. Kanter, Kaiser Permanente Southern California, Anaheim, California; Hilary K. Wall, Centers for Disease Control and Prevention, Atlanta, Georgia.

## Abstract

The Kaiser Permanente Southern California (Kaiser) health care system succeeded in improving hypertension control in a multiethnic population by adopting a series of changes in health care delivery. Data from the Healthcare Effectiveness Data and Information Set (HEDIS) was used to assess blood pressure control from 2004 through 2012. Hypertension control increased overall from 54% to 86% during that period, and 80% or more in every subgroup, regardless of race/ethnicity, preferred language, or type of health insurance plan. Health care delivery changes improved hypertension control across a large multiethnic population, which indicates that health care systems can achieve a clinical target goal of 70% for hypertension control in their populations.

## Objective

Hypertension affects about one-third of the US population (1) and is a major risk factor for heart attack and stroke (2,3). Million Hearts, an initiative launched by the US Department of Health and Human Services, aims to prevent 1 million heart attacks and strokes by 2017 through focused strategies, including improving blood pressure control (4). Million Hearts has a goal of 70% for blood pressure control in health care settings, which is achievable through changes in health care delivery (5). The objective of this study was to describe how health care delivery changes improved hypertension control in a multiethnic population.

## Methods

Kaiser Permanente Southern California (Kaiser) is a large integrated health care delivery system that provides care to approximately 3.5 million members in various inpatient and outpatient settings including 14 medical centers and about 200 medical offices (6). Since 2004, Kaiser has implemented a series of changes in health care delivery to improve identification and treatment of patients with hypertension and to increase hypertension control. These changes included the following: a validated hypertension population registry, a simple drug treatment algorithm based on a combination antihypertensive medication, regular performance feedback, alerts to providers that the patient has an elevated blood pressure measure, improved staff competency in blood pressure monitoring, expansion of patient access to primary care by using medical assistants to check blood pressure, and strong leadership support for health care delivery changes to improve hypertension control. Details on specific health care delivery changes have been published elsewhere (7).

The Healthcare Effectiveness Data and Information Set (HEDIS) is a tool for measuring performance in health care delivery and service in health plans in the United States (8). Using a common measure, such as a HEDIS measure, allows health care plans to report comparable estimates. The Controlling High Blood Pressure (CBP) measure is the percentage of adults aged 18 to 85 with a diagnosis of hypertension whose blood pressure was adequately controlled (<140/90 mm Hg) during the measurement year. Prior to 2006, CBP assessed hypertension only for adults aged 45 to 85. This article describes use of CBP to assess blood pressure control in Kaiser’s population and presents results from 2004 through 2012 as is done in the annual report of HEDIS data by the National Committee for Quality Assurance (9).

## Results

In 2012, Kaiser had more than 2 million members aged 18 years or older, of whom more than 400,000 had high blood pressure (Table 1). Most members with high blood pressure were aged 40 to 85, and about half were male (46.7%). About 46% of members with hypertension were non-Hispanic white and 26.6% were Hispanic. The remaining members were non-Hispanic black (14.9%) and non-Hispanic Asian (11.7%). One in 10 members reported Spanish as their preferred language (10.6%). Most of Kaiser’s patient population with high blood pressure had a commercial health insurance plan (52.7%) or Medicare (43.7%). Members with hypertension were older and more likely to be insured through Medicare or have diabetes compared with the overall Kaiser membership.

**Table 1:** Demographic Characteristics by Adult Membership, Hypertension Population, and the HEDIS Controlling High Blood Pressure Measure, Kaiser Permanente in Southern California (Kaiser), 2012

Demographic Characteristic	Kaiser Membership^a^, N (%)	Hypertension Population^b^, N (%)	Hypertension Control^c^, %
Total population	2,341,460	409,170 (17.5)	85.5
**Age, y**			
18–39	834,921 (35.7)	15,701 (3.8)	77.9
40–64	1,086,190 (46.4)	195,908 (47.9)	84.7
65–85	420,349 (17.9)	197,561 (48.3)	87.0
**Race/ethnicity**			
Hispanic	837,659 (35.8)	108,857 (26.6)	85.0
White, non-Hispanic	973,198 (41.6)	187,536 (45.8)	86.6
Black, non-Hispanic	235,108 (10.0)	60,938 (14.9)	81.4
Asian, non-Hispanic	267,196 (11.4)	47,874 (11.7)	87.9
Other, non-Hispanic	28,299 (1.2)	3,965 (1.0)	85.0
**Preferred language^d^**			
English	1,995,480 (85.2)	355,153 (86.8)	85.5
Spanish	248,965 (10.6)	43,564 (10.6)	85.7
**Sex**			
Male	1,104,124 (47.2)	190,933 (46.7)	85.9
Female	1,237,156 (52.8)	218,237 (53.3)	85.2
**Plan type^e^**			
Commercial	1,900,180 (81.2)	215,609 (52.7)	84.1
Medicare	376,614 (16.1)	178,718 (43.7)	87.2
Medicaid	33,731 (1.4)	5,587 (1.4)	84.6
**Comorbidity**			
Diabetes	252,398 (10.8)	115,530 (28.2)	86.4

Abbreviations: HEDIS, Healthcare Effectiveness Data and Information Set.

a Members ≥18 years

b Patients with hypertension as defined by HEDIS, aged 18 to 85 years (8).

c As measured by the HEDIS Controlling High Blood Pressure measure: patients with a diagnosis of hypertension (as defined by HEDIS) whose blood pressure was adequately controlled (<140/90 mm Hg) during the measurement year (8).

d Other preferred languages are not included.

e Other plan types are not included (eg, combined Medicare and Medicaid and self-insured).

As a result of sequential and concurrent changes in health care delivery from 2003 through 2010, blood pressure control increased to about 86% in the Kaiser patient population as measured by the HEDIS CBP measure. By 2012, blood pressure control was greater than 80% in all demographic groups with the exception of those aged 18 to 39, for whom the CBP measurement was 77.9%. By race/ethnicity, the CBP measurement ranged from 81.4% for non-Hispanic blacks to 87.9% for non-Hispanic Asians. Preferred language had little effect on blood pressure control: the CBP measure was 85.5% for English speakers and 85.7% for Spanish speakers. Members with commercial health insurance (84.1%), Medicare (87.2%), and Medicaid (84.6%) had similar CBP measurements.

Kaiser made health care delivery changes from 2003 through 2010 to improve hypertension control among members (Table 2). As care delivery changes were made, hypertension control increased. From 2004 through 2012, hypertension control increased from 54% to 86% as calculated by using the HEDIS CBP measurement.

**Table 2:** Timeline of Health Care Delivery Changes and the HEDIS Controlling High Blood Pressure Measure, Kaiser Permanente in Southern California, 2003–2012

Year	Hypertension Control^a^, %	Health Care Delivery Changes Introduced^b^
2003	—c	•National Kaiser Permanente hypertension treatment algorithm implemented •Plastic cards used to distribute new treatment algorithm
2004	54	•Hypertension population registry began •Hypertension identified as a chronic care condition •Monthly performance reports became available •Hypertension team-based care began •Diuretic-naïve program targeting uncontrolled hypertension patients began
2005	64	•Standard of care for new therapy was changed to use a fixed-dose combination drug •Regional hypertension leadership disseminated 5 best practices •Regional leadership began making hypertension a quality imperative •Standard of care became blood pressures taken in surgical specialty offices •Peer validator facilitated accurate blood pressure measurements •New furniture arrangements supported proper blood pressure measurement; large adult-sized cuffs purchased
2006	71	•Confirmation of initial blood pressure elevations used as a quality performance tool
2007	73	•Region-wide electronic health care record implemented •Electronic alerts replaced reminder cards •Electronic check list for out-of-control hypertension patients provided for medical assistants
2008	80	•Language concordance between physicians and patients increased; physicians tested for fluency
2009	83	•Medication refill adherence ratios and days of supply remaining became available to providers at point of care
2010	84	•Staff worked to full scope of practice •10-minute non-copayment visits with a medical assistant implemented for blood pressure check and triage according to a scope of practice protocol.
2011	86	—d
2012	86	—d

Abbreviations: HEDIS: Healthcare Effectiveness Data and Information Set; —, not applicable.

a As measured by the HEDIS Controlling High Blood Pressure measure: patients with diagnosis of hypertension (as defined by HEDIS) whose blood pressure was adequately controlled (<140/90 mm Hg) during the measurement year (8).

b Details on specific delivery changes have been published elsewhere (7).

c Data not available.

d No major changes in health care delivery for hypertension control were introduced in 2011 and 2012.

## Discussion

Changes in health care delivery improved hypertension control in a large and diverse population throughout all demographic subgroups, including Hispanic and non-Hispanic black patients and patients receiving Medicaid. The Kaiser health plan encompasses Los Angeles County, where 48% of residents are Hispanic compared with 38% in California overall and 17% in the United States (10). Nationally, there are significant gaps in awareness, treatment, and control of hypertension between the non-Hispanic white and Hispanic populations (1). However, there is no difference in hypertension control between these populations in the Kaiser population. Our results demonstrate that system-wide changes can affect all subpopulations of patients.

This study has several limitations. First, we described a continuous quality improvement process over time with many changes occurring simultaneously. The relative contribution of any of the individual best practices could not be quantified. Second, the hypertension registry may have included patients without hypertension. Finally, the importance of the context of a fully integrated health care delivery system cannot be estimated and probably magnified overall success. Therefore, these results may not be transferable to a system lacking integrated health care delivery.

Increasing hypertension control in the United States by 2017 to the Million Hearts population goal of 70%, including people with and without a usual source of care, would substantially reduce heart attacks and strokes (1). Treatment and control of hypertension has a greater impact in patients with other cardiovascular risk factors, such as age, black race, and additional comorbidities, such as diabetes (11,12). Through the implementation of a series of health care delivery changes, Kaiser improved hypertension control from 54% to 86% in its patient population. The systematic use of a collection of basic implementation tools and protocols is associated with significant success in hypertension control across a large multiethnic population.
